# Registration of PET and CT images based on multiresolution gradient of mutual information demons algorithm for positioning esophageal cancer patients

**DOI:** 10.1120/jacmp.v14i1.3931

**Published:** 2013-01-07

**Authors:** Shuo Jin, Dengwang Li, Hongjun Wang, Yong Yin

**Affiliations:** ^1^ School of Information Science and Engineering Shandong University Shandong China; ^2^ School of Physics and Electronics Science Shandong Normal University Shandong China; ^3^ Department of Radiation Oncology Shandong Tumor Hospital and Institute Shandong China

**Keywords:** ${\;}^{18}F-\text{FDG}$ PET‐CT images, deformation image registration, gradient of mutual information demons algorithm, modified Hausdorff distance, radiation therapy

## Abstract

Accurate registration of ${\;}^{18}F-\text{FDG}\;\text{PET}$ (positron emission tomography) and CT (computed tomography) images has important clinical significance in radiation oncology. PET and CT images are acquired from ${\;}^{18}F-\text{FDG}$ PET/CT scanner, but the two acquisition processes are separate and take a long time. As a result, there are position errors in global and deformable errors in local caused by respiratory movement or organ peristalsis. The purpose of this work was to implement and validate a deformable CT to PET image registration method in esophageal cancer to eventually facilitate accurate positioning the tumor target on CT, and improve the accuracy of radiation therapy. Global registration was firstly utilized to preprocess position errors between PET and CT images, achieving the purpose of aligning these two images on the whole. Demons algorithm, based on optical flow field, has the features of fast process speed and high accuracy, and the gradient of mutual information‐based demons (GMI demons) algorithm adds an additional external force based on the gradient of mutual information (GMI) between two images, which is suitable for multimodality images registration. In this paper, GMI demons algorithm was used to achieve local deformable registration of PET and CT images, which can effectively reduce errors between internal organs. In addition, to speed up the registration process, maintain its robustness, and avoid the local extremum, multiresolution image pyramid structure was used before deformable registration. By quantitatively and qualitatively analyzing cases with esophageal cancer, the registration scheme proposed in this paper can improve registration accuracy and speed, which is helpful for precisely positioning tumor target and developing the radiation treatment planning in clinical radiation therapy application.

PACS numbers: 87.57.nj, 87.57.Q‐, 87.57.uk

## I. INTRODUCTION

Currently, the efficiency of radiation therapy mostly depends on the accuracy of positioning the tumor and completely identifying the potential tumor tissue.^(^ ^1^ ^,^ ^2^ ^)^ As the development of different imaging technology, the application of multimodality imaging has already addressed many problems, making the accurate positioning tumor and tracking the tumor motion to be possible. In clinical diagnosis, the patients with suspected cancerous lesions often undergo multiple examinations using several modalities, then image process techniques are used to improve the accuracy of clinical images.^(^ ^3^ ^,^ ^4^ ^)^ Multimodality images can reflect tumor states according to different imaging principle, and these noninvasive imaging technologies can be divided into two types — anatomical and functional images. Anatomical imaging provides structural information images with high‐resolution, such as computed tomography (CT), magnetic resonance (MR), and ultrasound (US); however, functional imaging provides human body functional metabolism information images with coarser resolution, such as positron emission tomography (PET), single‐photon emission computed tomography (SPECT), and functional magnetic resonance imaging (FMRI).^(^ ^5^ ^)^ The information provided by single modality is insufficient, which can be solved by different imaging systems. Complementary modalities by integrating anatomical and functional datasets are used in clinical examinations, and the images from complementary modalities can facilitate the detection and identification of suspected cancerous lesions.

${\;}^{18}F-\text{FDG}$ PET‐CT imaging modality combines whole‐body anatomical and functional information. For PET imaging system, as ${\;}^{18}F-\text{Fluorodeoxyglucose}$ (${\;}^{18}F-\text{FDG}$) for tracer, PET equipment is used to detect the high‐energy γ photon generated by ${\;}^{18}F$, so PET images can provide information of ${\;}^{18}F-\text{FDG}$ metabolism, which can clearly display high glucose metabolism with lesion, but anatomic structures in PET image are not clear. However, CT images, with high‐image resolution, can show anatomic structures, which is helpful for accurate positioning abnormal metabolism region in PET images. Therefore, PET‐CT imaging modality fundamentally overcomes the shortcoming of unclear anatomic structure in nuclear medical image, and nuclear medical image is whole energy attenuation corrected by CT image, which reached the purpose of quantitative. Compared with separate PET and CT system, ${\;}^{18}F-\text{FDG}$ PET‐CT imaging system has high sensitivity and specificity for diagnosing cancer, which is widely used in radiation therapy, and has a significant value for precisely positioning tumor, estimating lesion property, delineating the tumor target, and evaluating the radiotherapy effects.^(^ ^6^ ^–^ ^8^ ^)^

For current whole‐body ${\;}^{18}F-\text{FDG}$ PET‐CT scanning technique, the acquisition and integration of the two image datasets are still performed separately, and the whole acquisition process takes a long time, so every part of the body, especially viscera and respiratory‐related muscles, cannot be held stationary for such a long time. In order to accurately reflect the information of corresponding points between PET and CT images, it is necessary to register the two images before fusion. For multimodality images, the most common registration method was based on mutual information (MI), which has been confirmed as the most basic method to match PET and CT images.^(^ ^4^ ^,^ ^9^ ^,^ ^10^ ^)^ Rigid transformation was used to automatically register whole‐body PET‐CT‐SPECT; however, the local deformation cannot be solved.^(^ ^11^ ^)^ A new 3D elastic transformation algorithm based on normalized mutual information (NMI) was used to automatically match PET and CT images. This method showed better precision compared with rigid transformation.^(^ ^12^ ^)^ In the study by Suh et al.,^(^ ^13^ ^)^ the authors presented a new deformable method based on a weighted demons algorithm to register whole‐body rat CT and PET images registration, and they used the maximum likelihood Hausdorff distance as similarity measure. The results demonstrated higher property in comparison with traditional demons algorithm and NMI‐based nonrigid free‐form deformation (FFD) method.

The quantitative assessment of registration results is a major challenge, especially multimodality images registration. Candidate metrics, used for assessing the performance of registration results, include root mean squared error, normalized crosscorrelation, similarity coefficient, or volume overlap index (VOI), along with Hausdorff distance (HD), mutual information (MI), and average surface‐to‐surface distance.^(^ ^14^ ^)^ PET and CT images are acquired from different imaging equipment, but they are based on the same human anatomical information. In order to quantify the spatial accuracy of different registration methods, we choose modified Hausdorff distance (M‐HD) as the performance metric to evaluate the results of different image registration algorithms.^(^ ^15^ ^,^ ^16^ ^)^

In this paper, we have implemented and validated a deformable CT to ${\;}^{18}F-\text{FDG}\;\text{PET}$ image registration method for locally advanced esophageal cancer. Treating PET image as the reference image, the corresponding CT image is used as the floating image. Firstly, we preprocess PET and CT images by global registration method, minimizing margin errors in global; then register local deformation between PET and CT images using GMI demons algorithm. The accuracy of the algorithm was evaluated by M‐HD. In addition to speeding up the registration process, maintain its robustness, and avoid the local extremum, the multiresolution image pyramid structure was used before deformable registration. By quantitative and qualitative analysis, the registration scheme used in this paper has clinical value of accurately positioning tumor target, which was helpful for the next clinical radiotherapy application.

## II. MATERIALS AND METHODS

Ten locally advanced esophageal cancer patients were PET/CT simulated. Before the PET examination, all patients fasted for at least 6 h, and each patient's blood glucose level was be measured. Patients did not undergo urinary bladder catheterization and received no oral muscle relaxants. ${\;}^{18}F-\text{FDG}$ PET/CT scans were obtained with an advanced PET/CT scanner (Discovery LS; GE Healthcare, Waukesha, WI). Sixty minutes after intravenous injection of 370 MBq (10mCi) of ${\;}^{18}F-\text{FDG}$, emission scans were obtained from head to thigh for 5 min per field of view, each covering 14.5 cm, at an axial sampling thickness of 4.25 mm/slice. (The slice thickness of PET and CT images are 4.25 mm.) The PET/CT system was used for four‐slice helical CT acquisition, followed by a full‐ring dedicated PET scan of the same axial range. The CT component was operated with an X‐ray tube voltage peak of 120 keV, 90 mA, a 6:1 pitch, a slice thickness of 4.25 mm, and a rotational speed of 0.8 s/rotation. Both the PET scans and the CT scans were obtained during normal breathing. PET images were reconstructed with CT‐derived attenuation correction using ordered‐subset expectation maximization software. The attenuation‐corrected PET images, CT images, and fused PET/CT images were available for review in axial, coronal, and sagittal planes, as was a cine display of maximum intensity projections of the PET data, using the manufacturer's review station (Xeleris; GE Healthcare).

### A. demons algorithm

Demons algorithm is proposed by Jean‐Philippe Thirion, which is similar with the experiment principle of Maxwell.^(^ ^17^ ^)^ Since its establishment, this algorithm has already successfully been applied in medical image registration.^(^ ^18^ ^–^ ^20^ ^)^ The basic idea of demons algorithm is to simulate image registration as a diffusion process, where contours in the reference image are treated as semipermeable membranes (SMs) and the floating image diffuse into or out of the SMs by the actions of “demons” situated in the SMs. Depending on factors such as the selection of the demon positions and calculation of the force of a demon, many variants of the demons algorithm have been created and implemented.

This method drives pixels in floating image to move by judging the motion direction of every pixel, and the “demons” force of every pixel drives floating image to register with reference image, achieving the purpose of nonrigid registration. The gradient $\bar{\nabla }r$ of reference image is internal force of “demons” force, and the grayscale difference of corresponding pixels between the two images is external force. For fixed point P, *r* is the grayscale of reference image R, and *f* is of floating image F. The offset for fixed point P between two images is calculated according to:

(1) $$\vec{v}=\frac{(f-r)\times \vec{\nabla }r}{||\vec{\nabla }r|{|}^{2}+\frac{{\left(f-r\right)}^{2}}{k^{2}}}$$

where *k* is a normalization factor to compensate for the mismatch between two images.

Since demons algorithm utilizes local information to transform images, in order to make the transformation continuously in global scope and maintain the image topology, demons algorithm iteratively updates the offset using the following equation for every pixel:

(2) $${\vec{v}}_{n+1}=G_{\sigma }*({\vec{v}}_{n}+\frac{(f-r)\times \vec{\nabla }r}{||\vec{\nabla }r|{|}^{2}+\frac{{\left(f-r\right)}^{2}}{k^{2}}}$$

where $G_{\sigma }$ is the Gaussian filter with a standard deviation σ, which is used to smooth the offset between iterations to regularize the deformation.

Based on demons algorithm to register two images, grayscale difference of corresponding pixels is treated as the external force. For floating image, the pixels with same grayscale will be mapped to one point in reference image, so the topology structure of floating image will be changed, and misalign will be occurred between two images. Moreover, in the absence of image gradient information, mismatch is easily caused. Due to the disadvantage of demons method, GMI demons algorithm is used in this paper, which can register correctly when image gradient is zero.

### B. Gradient of mutual information based demons algorithm

Demons algorithm, based on optical flow field, has the advantages with fast process speed and high accuracy, and this traditional demons method drive floating image to transform only depends on grayscale difference and grayscale gradient of reference image. However, multimodality images come from different modal imaging device; the inherent imaging mechanism decide that grayscale distribution vary greatly, so demons algorithm could not transform images correctly, and is only suitable for single‐modality image registration.^(^ ^21^ ^)^ In addition, when $\left\|\vec{\nabla }r\right\|\to 0$, the movement direction of pixel cannot be determined, and the deformation direction of floating image also cannot be determined, which can easily cause image misalignment.

Based on the advantages of demons algorithm, and combined MI method which can match multimodality images, the gradient of mutual information‐based demons (GMI demons) algorithm is proposed in this paper, adding an additional external force based on the gradient of mutual information (GMI) between two images, which can achieve PET and CT images deformable registration with esophageal cancer.

The GMI demons algorithm based on the gradient of mutual information is calculated according to:

(3) $${\vec{v}}_{n+1}=G_{\sigma }*({\vec{v}}_{n}+\frac{(f-r)\times \vec{\nabla }r}{||\vec{\nabla }r|{|}^{2}+\frac{{\left(f-r\right)}^{2}}{k^{2}}}+\alpha \vec{\nabla }MI({\vec{v}}_{n}))$$

For a fixed point P, $\vec{\nabla }MI({\vec{v}}_{n})$) is the gradient of mutual information between PET and CT images for current transformation, and is a positive constant, representing the iteration step.

For a fixed point P, *i* is the grayscale of fixed point P in reference image, and *j* is of the point $P+\vec{v}$ in floating image, the derivative of mutual information to current spatial displacement vector is defined as the gradient of mutual information, and rewritten joint distribution $P_{FR}(i,j)$ of image grey as a continuous function by Parzen window.

(4) $$MI(\vec{v})=\iint p_{\bar{v}}^{RF}(i,j)\log\frac{p_{\bar{v}}^{RF}(i,j)}{p^{R}(i)p_{\bar{v}}^{F}(j)}didj$$

The joint entropy distribution of image grayscale is estimated by the number of overlap pixels N between two images. The 2D Parzen window with the width of δ is chosen to use in the estimation process:

(5) $$p_{\bar{v}}^{RF}(i,j){=}_{N}^{1}\int_{\omega }\Psi_{\delta }(R(P)-i,F(P+\vec{v})-j)dP$$

where $\Psi_{\delta }(i,j)=K_{\delta }(i)K_{\delta }(j),K_{\delta }(t)=\frac{1}{\sqrt{2\pi \delta }}\exp(-\frac{t^{2}}{2\delta^{2}})$.

The gradient of mutual information $\vec{\nabla }MI(\vec{v})$ can be calculated according to:

(6) $$\vec{\nabla }MI(\vec{v})=\frac{1}{N}[\psi_{\delta }⊗\frac{dL_{\vec{v}}}{dj}](R(P)F(P+\vec{v}))\vec{\nabla }F(P+\vec{v})$$

where $L_{\vec{v}}(i,j)=1+\log\frac{p_{\vec{v}}^{RF}(i,j)}{p^{R}(i)p_{\vec{v}}^{F}(j)}$ and $\vec{\nabla }F(P+\vec{v})$ is defined as the grayscale gradient of point $P+\vec{v}$ in floating image.

### C. Registration process

**Figure 1:**
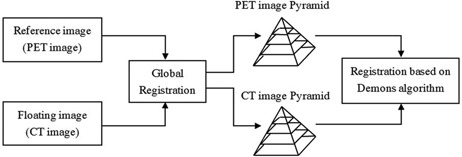
Flow chart of multiresolution demons algorithm to register CT images with PET images.

To speed up the registration process, maintain its robustness. and avoid the local extremum, a multiresolution technique was used in our study.^(^ ^22^ ^)^ As shown in Fig. 1, the original PET and CT images were down‐sampled successively, forming two image pyramids, and multiresolution image registration is achieved iteratively from the coarsest level to finest level. The registration process is realized by these steps:

1. Preprocess PET and CT images by a global registration to minimize the margin error, and achieve the alignment of these two images on the whole.
2. For the floating image (CT image), set the initial offset of position vector ${\vec{x}}_{i}$ as ${\vec{v}}_{i}=0$ for every pixel *i*, then form PET and CT image pyramids by down‐sampling these two images successively, and *k* is the pyramid level.
3. At the current resolution level, register PET and CT images by GMI demons algorithm. After *n* iterations, the position vector ${\vec{x}}_{i}^{n}$ of pixel *i* in floating image is calculated by ${\vec{x}}_{i}^{n}={\vec{x}}_{i}^{n-1}+{\vec{v}}_{i}^{n-1}$ can be calculated according to Eq. (3).
4. Estimate ${\vec{v}}_{i}^{n}$, if the increment of MI between two images falls below a specific threshold, the iteration at current resolution level is considered to have reached a stable state, enter into next resolution level to registration. If not, return to n+1 iteration (1≤n<N).
5. The next resolution level is formed by oversampling the current resolution level, and the offset transformation of current level is set as initial value of next level, return to step 3 to iterate.
6. Multiresolution image registration is achieved iteratively from coarse to fine, stepwise refinement, and the offset transformation at each resolution level is obtained by GMI demons algorithm. After all the resolution levels are accomplished, acting the ultimate offset transformation on the floating image, reach the purpose of precise registration of PET and CT images.

### D. Modified Hausdorff distance

Hausdorff distance can reflect the maximum no‐matching degree between two images. When the HD value is bigger, it means the difference between the two images is greater. For discrete digital images A and B, the Hausdorff distance is defined as:

(7) H(A,B)=max(h(A,B),h(B,A))

where H(A,B) and H(B,A) are the directed Hausdorff distances of A→B  and B→A, respectively, and they are defined as:

(8) $$h(A,B)=\underset{a\in A}{\max}d(a,B)=\underset{a_{i}\in A}{\max}\;\underset{b_{j}\in B}{\min}||a_{i}-b_{j}||$$

(9) $$h(B,A)=\underset{b\in B}{\max}d(b,A)=\underset{b_{i}\in B}{\max}\;\underset{a_{j}\in A}{\min}\left\|b_{j}-a_{i}\right\|$$

where ||·|| is some distance norm in the plane, which we here restrict to being the Euclidean distance metric.

The traditional Hausdorff distance is easy to implement in calculation, but is sensitive to noise and cover, so we choose the modified Hausdorff distance (M‐HD), proposed by Dubuisson and Jain.^(^ ^16^ ^)^ The M‐HD can avoid the deviation caused by the interference of some noise pixels. h(A,B) is defined as:

(10) $$h_{MHD}(A,B)=\frac{1}{N_{a}}\sum_{a\in A}d(a,B)$$

where $N_{A}$ is the number of feature points in point set A.

## III. RESULTS

We report results of PET and CT images registration on a total of ten clinically acquired whole‐body PET/CT image pairs with esophageal cancer. Original datasets for the experiments came from the clinical cases archived at Shandong Tumor Hospital. The image data were picked by physician not familiar with algorithm of PET and CT image registration, only on the basis of availability of the multimodality images, without any other screening criteria, from a specific clinical condition to prognosis status.

**Figure 2:**
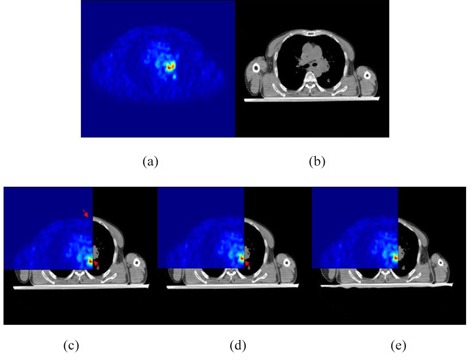
Split window display of PET and CT images: (a) PET image; (b) CT image; (c) overlaid PET‐CT image without any registration; (d) overlaid PET‐CT image with global registration; (e) overlaid PET‐CT image with GMI demons method registration. After precise registration, the two images match each other well. Red arrows indicate the places of larger errors.

In current clinical application, physicians will delineate tumor target in CT images based on PET images, which include GTV (gross tumor volume) and PTV (planning target volume). Then the physicist will outline endanger organs and choose appropriate radiotherapy mode to simulate radiation field and radiation dose. By generating isodose chart and analyzing DVH (dose‐volume histogram), physicist would develop accurate radiation treatment planning, which will improve radiotherapy effects. As shown in Fig. 2, (a) and (b) are PET and CT images, respectively, (c) to (e) are overlaid PET‐CT images displayed by split windows. As shown in Fig. 2(c), before registration there are obvious global and local errors between PET and CT images, which would affect the accuracy of radiation treatment planning and the radiotherapy effects. As shown in Fig. 2(d), after global registration, the two images match each other on the whole, but there are still obvious errors in local. As shown in Fig. 2(e), after GMI demons algorithm registration, PET and CT images register each other very well, which is helpful for doctors to identify tumor volume based on PET/CT images, and for physicists to develop radiation treatment planning. Through PET and CT images registration, we can accurately position tumor volume and reduce tumor target area, meanwhile reducing extended range of tumor volume by physicians independently, which ensures high doses of radiation within tumor area, and is not harm to adjacent tissues and organs, and achieves the purpose of precise radiotherapy.

**Figure 3:**
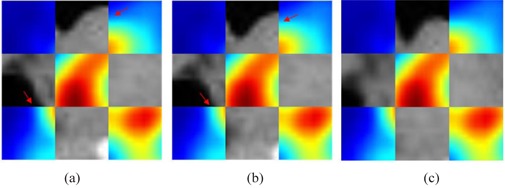
Checkerboard display of ${\;}^{18}F-\text{FDG}\;\text{PET}$ images with high metabolic lesion region and CT images: (a) before registration; (b) after global registration; (c) after GMI demons algorithm registration.

As shown in Fig. 3, according to the high metabolic lesion region in ${\;}^{18}F-\text{FDG}\;\text{PET}$ image, we can easily identify and locate tumor position, but physicians develop radiotherapy planning only in CT images, so precise and rapid registration of PET and CT images is a far‐reaching work. As shown in Fig. 3(a), there are serious errors between PET and CT images; therefore, manually outlining tumor volume will certainly lead to the larger tumor target area, harming patients' normal tissues and organs and affecting the radiation efficiency. As shown in Fig. 3(b), after global rigid registration, errors between PET and CT images is smaller, but there is still misalignment, and the accuracy is far from practical requirements. As shown in Fig. 3(c), after GMI demons‐based deformable registration, the result is demonstrated to be accurate through evaluation of physician, achieving the purpose of accurate positioning tumor, with great clinical significance.

For multimodality images, the most common registration algorithm is based on MI; after MI‐based deformable registration, multimodality images are regarded as accurate match. Table 1 shows the M‐HD similarity measures before and after different registration algorithms for ten esophageal cancer patients. The purpose of Table 1 is to contrast the spatial accuracy of two images before registration, after global rigid registration, after MI‐based deformable registration, and after GMI demons‐based deformable registration. As shown in Table 1, the values of M‐HD between PET and CT images after global rigid registration are less than the initial images; and the M‐HDs after deformable registration are less than the values after global rigid registration; and for deformable registration, the M‐HD values after GMI demons‐based deformable registration are less than that after MI‐based deformable registration. Based on the advantages of demons algorithm and combined MI method which can match PET and CT images, the GMI demons algorithm can realize PET and CT images deformable registration, and this method performs better to improve the accuracy of registration.

**Table 1:** Comparison of M‐Hausdorff distance before and after registration for ten esophageal cancer cases.

			*Deformable Registration*		
	*Before Registration*	*After Global Registration*	*After MI Registration & Improved Ratio/%*	*After GMI Demons Registration & Improved Ratio/%*	*Ratio Difference (Δ/%)*
Patient 1	3.972	3.427	2.906 & 26.84	2.569 & 35.32	8.48
Patient 2	6.137	5.105	4.238 & 30.94	3.758 & 38.76	7.82
Patient 3	8.617	7.236	6.175 & 28.34	5.988 & 30.51	2.17
Patient 4	9.863	8.549	7.478 & 24.18	7.039 & 28.63	4.45
Patient 5	4.736	4.215	3.842 & 18.88	3.654 & 22.85	3.97
Patient 6	7.326	6.087	5.301 & 27.64	4.900 & 33.11	5.47
Patient 7	7.975	6.983	6.075 & 23.82	5.628 & 29.43	5.61
Patient 8	5.648	5.273	4.887 & 13.47	4.308 & 23.73	10.26
Patient 9	6.794	5.937	4.854 & 28.55	4.520 & 33.47	4.92
Patient 10	9.271	8.481	7.289 & 21.38	6.580 & 29.03	7.65

## IV. DISCUSSION

Multimodality medical image registration is always a difficult work for clinical applications, while MI‐based registration as the most common algorithm for multimodality images is now by far the preferred method for clinical use. As shown in Table 1, the registration results based on GMI demons algorithm is much better than that based on MI method. The GMI demons algorithm, combined the advantages of demons algorithm and the gradient of mutual information, is suitable for the image registration from different imaging systems. Our paper uses this algorithm to match ${\;}^{18}F-\text{FDG}\;\text{PET}$ and CT images with esophageal cancer, improving the registration accuracy and the values of clinical application.

**Figure 4:**
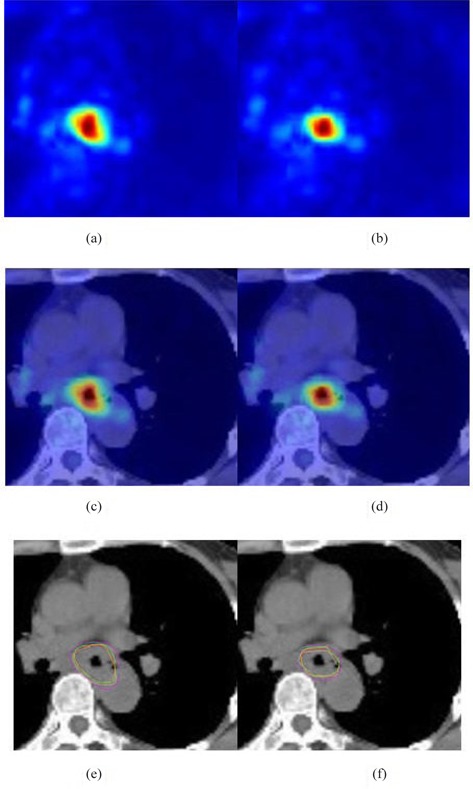
Comparison of GTV before and after GMI demons method‐based registration: PET images (a) before and (b) after registration; PET‐CT fusion images (c) before and (d) after registration; delineation of GTV in CT images based on (e) PET and (f) PET‐CT fusion images. Five circles with different colors represent the GTV contours of five physicians.

${\;}^{18}F-\text{FDG}\;\text{PET}$‐CT body scanning technology, combined the information of function and anatomy imaging, has the particular sensitivity to detect cancer in the early stage that other devices cannot. The challenge in implementing tumor radiation therapy based on PET and CT images is semiautomatic or automatic GTV delineation, which often relies on efficient and robust image registration. For patients with esophageal cancer, the cancerous part is in the chest center and there are many important tissues and organs around such as lung, heart, and spine. Accurately positioning tumor target can not only ensure the safety of surrounding normal tissues and organs, but also improve the radiation dose within tumor target area as much as possible, better affecting the radiation therapy. As shown in Fig. 4, five physicians delineate the GTVs in CT image according to corresponding PET image. And after GMI demons‐based registration method, the GTV areas are obviously less than that before registration — that is to say, the new GTV after registration not only includes all tumor area, but also removes the extended area caused by various errors, which has significant values in clinical application.

The GTVs of ten esophageal cancer patients were separately delineated by five physicians, and the mean and standard deviation of the GTVs before and after registration are summarized in Table 2. As shown in Table 2, after MI‐based registration, the GTV is less than before registration, and after GMI demons‐based registration that is less than MI‐based, and the Δ of improved ratio between two registration methods is consistent with the Δ of M‐HDs in Table 1. So developing radiotherapy planning based on GTV after accurate registration will definitely reduce the radiation quantity of surrounding normal tissues and organs, and increase the absorbed doses within tumor, which demonstrates that accurately registering PET and CT images will improve the efficiency of tumor radiation therapy, and has an important research meaning and clinical value for tumor treatment.

**Table 2:** Mean GTV (${\text{cm}}^{3}$) for ten esophageal cancer patients delineated by five physicians before and after registration.

	*GTV(SD) Before Registration*	*GTV(SD) after MI Registration & Improved Ratio/%*	*GTV(SD) after GMI Demons Registration & Improved Ratio/%*	*Ratio Difference (Δ/%)*
Patient 1	3.34 (0.08)	2.48(0.03) & 25.75	2.20(0.09) & 34.13	8.38
Patient 2	6.80(0.12)	4.57(0.14) & 32.79	4.05(0.09) & 40.44	7.65
Patient 3	17.47(0.23)	12.57(0.17) & 28.05	12.18(0.27) & 30.28	2.23
Patient 4	21.38(1.00)	16.52(0.22) & 22.73	15.53(0.21) & 27.36	4.63
Patient 5	4.87(0.09)	3.88(0.02) & 20.33	3.69(0.06) & 24.23	3.90
Patient 6	9.19(0.15)	6.73(0.09) & 26.66	6.25(0.07) & 32.00	5.34
Patient 7	15.50(0.19)	11.93(0.21) & 23.03	11.08(0.28) & 28.52	5.49
Patient 8	5.81(0.02)	5.08(0.04) & 12.56	4.49(0.01) & 22.72	10.16
Patient 9	7.98(0.02)	5.73(0.06) & 28.20	5.35(0.03) & 32.96	4.76
Patient 10	20.83(0.09)	16.30(0.10) & 21.75	14.74(0.06) & 29.24	7.49

## V. CONCLUSIONS

In this work, we have implemented a deformable image registration method to match CT image to corresponding ${\;}^{18}F-\text{FDG}\;\text{PET}$ image with locally advanced esophageal cancer. The accuracy of the registration algorithm was quantified by M‐HD. The values of M‐HD between PET and CT images, calculated before registration, after global registration, after MI‐based deformable registration, and after GMI Demons‐based deformable registration, indicated that GMI demons algorithm has access to higher registration accuracy. By contrasting the changes of mean GTV between different methods, the registration scheme used in this paper is a promising tool to implement accurate positioning GTV for radiation therapy.

## ACKNOWLEDGMENTS

This work is jointly supported by the Shandong Natural Science Foundation (ZR2010HM010 and ZR2010HM071), and also supported by the Project of Shandong Province Higher Educational Science and Technology Program (No.J12LN23), Research Fund for Excellent Young, Middle‐aged Scientists of Shandong Province (No.BS2012DX038), China Postdoctoral Science Foundation (No.2012M511538), Post‐doctoral Innovation Fund of Shandong province (201202032), and National Natural Science Foundation of China (No.61201441).
